# Immuno-Modulation of Hematopoietic Stem and Progenitor Cells in Inflammation

**DOI:** 10.3389/fimmu.2020.585367

**Published:** 2020-11-24

**Authors:** Maiko Sezaki, Yoshikazu Hayashi, Yuxin Wang, Alban Johansson, Terumasa Umemoto, Hitoshi Takizawa

**Affiliations:** ^1^ Laboratory of Stem Cell Stress, International Research Center for Medical Sciences (IRCMS), Kumamoto University, Kumamoto, Japan; ^2^ Laboratory of Hematopoietic Stem Cell Engineering, International Research Center for Medical Sciences (IRCMS), Kumamoto University, Kumamoto, Japan; ^3^ Division of Functional Structure, Department of Morphological Biology, Fukuoka Dental College, Fukuoka, Japan; ^4^ Department of Hematology, Zhujiang Hospital, Southern Medical University, Guangzhou, China; ^5^ Center for Metabolic Regulation of Healthy Aging, Kumamoto University, Kumamoto, Japan

**Keywords:** hematopoietic stem cells, BM environment, inflammation, infection, immune-memory

## Abstract

Lifelong blood production is maintained by bone marrow (BM)-residing hematopoietic stem cells (HSCs) that are defined by two special properties: multipotency and self-renewal. Since dysregulation of either may lead to a differentiation block or extensive proliferation causing dysplasia or neoplasia, the genomic integrity and cellular function of HSCs must be tightly controlled and preserved by cell-intrinsic programs and cell-extrinsic environmental factors of the BM. The BM had been long regarded an immune-privileged organ shielded from immune insults and inflammation, and was thereby assumed to provide HSCs and immune cells with a protective environment to ensure blood and immune homeostasis. Recently, accumulating evidence suggests that hemato-immune challenges such as autoimmunity, inflammation or infection elicit a broad spectrum of immunological reactions in the BM, and in turn, influence the function of HSCs and BM environmental cells. Moreover, in analogy with the emerging concept of “trained immunity”, certain infection-associated stimuli are able to train HSCs and progenitors to produce mature immune cells with enhanced responsiveness to subsequent challenges, and in some cases, form an inflammatory or infectious memory in HSCs themselves. In this review, we will introduce recent findings on HSC and hematopoietic regulation upon exposure to various hemato-immune stimuli and discuss how these challenges can elicit either beneficial or detrimental outcomes on HSCs and the hemato-immune system, as well as their relevance to aging and hematologic malignancies.

## Cellular Heterogeneity in Early Hematopoiesis and the BM Niche in Steady State

Lifelong replenishment of all mature blood and immune cells is sustained by a rare population of hematopoietic stem cells (HSCs) through a hierarchically organized lineage commitment program. In steady-state, the adult HSC pool is relatively quiescent but upon cell cycle entry, a stepwise specification of long-term reconstituting HSCs to progressively multi-, oligo- and uni-potent hematopoietic progenitors (HPCs) restricted to the myeloid, lymphoid, and megakaryocyte-erythroid lineages supply the total blood cell pool (1). This program shows flexibility and durability to sudden hematopoietic perturbations such as blood loss or inflammation and reflects strict control over HSC self-renewal versus differentiation, as exhaustion or an imbalance in either will readily amount in hematopoietic failure and/or hematologic malignancies. The section will review current findings on HSC heterogeneity and its contribution toward steady-state hematopoiesis and briefly, cover essential concepts of the BM niche relevant later in the text for understanding the impact of a perturbed or stressed BM environment on HSCs.

The recent advancement in single-cell-based techniques and analysis (e.g., single-cell transplantation, RNA/ATAC-sequencing) has been revealing in terms of HSC biology in both native and stress hematopoiesis (2). The traditional roadmap of hematopoiesis, where HSCs were once assumed homogeneous with identical differentiation ability is currently being reassessed. The HSC population is in fact heterogeneous as clarified from single-cell transplantation and lineage-tracing experiments with certain HSC subsets being biased toward either myeloid or lymphoid lineages.

A large pool of multipotent progenitors (MPPs) rather than HSCs has been thought to drive steady-state hematopoiesis. Supportive of this are several publications that utilize lineage-tracing of genetically-labeled HSCs and barcoding *via* transposon tagging (3, 4). In contrast, Sawai et al., report of *Pdzk1ip1*-GFP-labeled HSCs as the ultimate source of continuous lymphopoiesis and myelopoiesis under steady-state conditions, which can be accelerated by a systemic interferon response (5). Chapple et al., utilize two independent HSC tracers, *Krt18* and *Fgd5* to likewise support this model, and additionally report a robust HSC contribution toward platelet and myeloid lineages (6). Although with no lineage bias as described in the previous study, Lu et al., similarly claim all donor-derived HSC clones regenerate the blood homogeneously under homeostasis, while in perturbed or conditioned states, a small fraction of engrafted HSC clones will dominantly expand and exhibit lineage bias (7). While this issue remains unresolved, the heterogeneity of HSCs nonetheless adds an additional layer of complexity in understanding HSC biology and requires consideration when interpreting functional readouts of steady-state and stress hematopoiesis, including infection and inflammation.

In addition to the well-established HSC immunophenotypes, lineage^-^Sca-1^+^c-Kit^+^ (LSK), endothelial protein C (EPCR) (8), and the SLAM family proteins (9) used for the isolation of phenotypic hematopoietic stem and multipotent progenitors (HSPCs), others have been described to reflect HSC function by enriching for distinct lineage bias upon transplantation. Recently, Neogenin-1 (NEO1) was identified to distinguish NEO1^+^ HSCs primed toward myelopoiesis at the cost of lymphopoiesis from NEO1^-^ HSCs that show a balanced differentiation into both myelopoiesis and lymphopoiesis (10). NEO1^+^ *Hoxb5* ^+^ HSCs expand with age while NEO1^-^ *Hoxb5* ^+^ HSC counts remain unchanged as in young mice. Additionally, in vWF-GFP reporter mice, the megakaryocytic gene involved in platelet aggregation encoding von Willebrand factor (vWF) (11) was found to be expressed in ~60% of phenotypically defined HSCs (LSKCD150^+^CD48^-^CD34^-^) with a higher expression of Mpl, encoding the thrombopoietin (TPO) receptor essential for megakaryocyte (Mk) and platelet production (12). This platelet-primed HSC subset with myeloid bias gives rise to lymphoid-biased HSCs, and thus are considered higher up in the hierarchical tree (11). The deficiency of Mitofusin 2, a regulator for mitochondrial fusion and tethering to the endoplasmic reticulum, results in reduced differentiation potential toward the lymphoid lineages (13). Several reports claim that platelet-biased HSCs residing at the apex of the HSC hierarchy are a major contributor of daily platelet production (14, 15). Indeed, a population of stem-like Mk-committed progenitors, primed but quiescent during steady-state and activated only during acute inflammation to replenish the depleted platelet pool has also been reported (16). Lineage skewing of HSCs toward platelets has been similarly observed in the BM of aged mice, where dysfunctional aged macrophages with an enhanced inflammatory signature fail to efficiently clear presiding apoptotic cells. The resulting increase in IL-1β is thought to induce the observed megakaryocytic HSC bias (17). Mks as a distinct lineage segregated from other hematopoietic lineages is so far implicated from several studies. HSC subtypes briefly noted here have been ably reviewed by Yamamoto et al., in which they discuss how emerging concepts of HSC heterogeneity presented *via* recent platelet and red blood cell lineage analyses may require a redefining of the “stemness” concept (18).

HSCs reside within the BM niche, a myriad of cellular, molecular and physical components of the BM microenvironment that maintain HSCs through the release of certain niche factors (19, 20). The perivascular niche has been well-described and is comprised of endothelial cells (ECs) and CXCL12 abundant reticular (CAR) cells, leptin receptor (LepR)^+^ cells, and nestin^+^ cells, with the latter three showing considerable overlap and high expression levels of stem cell factor (SCF) and CXCL12. Niche constituents are crucial in regulating HSC identity as demonstrated by several deletion studies. For example, SCF deletion in LepR^+^ cells and ECs eliminates quiescent and transplantable HSCs from the BM (21). Depletion of Mks (22, 23) and periarteriolar NG2^+^ stromal cells (24) results in HSC proliferation and are likewise thought to promote HSC quiescence. Interestingly, lineage-biased HSCs appear to occupy distinct BM microenvironments. Recently, myeloid-biased vWF^+^ HSCs were found to be enriched in Mk niches, while lymphoid-biased vWF^-^ HSCs were situated near quiescence-regulating arteriolar niches (25). Similar to HSCs, niche components are heterogeneous and form complex microenvironments with multiple inputs from cellular constituents. Together, a combination of cells coordinates the maintenance of the hematopoietic system both during steady-state and under perturbed situations.

## Inflammation-Stressed Early Hematopoiesis

Inflammation is the physiological reaction of the body to tissue injury or foreign insult and triggers a protective response involving blood and immune cells, vessels, and various molecular mediators. This is best illustrated in the case of infection; immune cells at local sites are activated through self or non–self-antigen recognition, and subsequent waves of innate and acquired immunity are coordinated to ensure host defense (26). In contrast to secondary lymphoid organs primarily tasked with immune activation, primary lymphoid organs including the BM had been long regarded immune-privileged with only minor exposure to the immune response. BM-residing HSCs and memory immune cells were thus assumed exempt from immune insults that can cause cell exhaustion or death, and reserved for prospective life-threatening invasions. HSCs were considered safely shielded in a dormant state through transcriptional and epigenetic regulators and their role in the initiation and resolution of inflammatory insults was presumed minimal.

**Figure 1:**
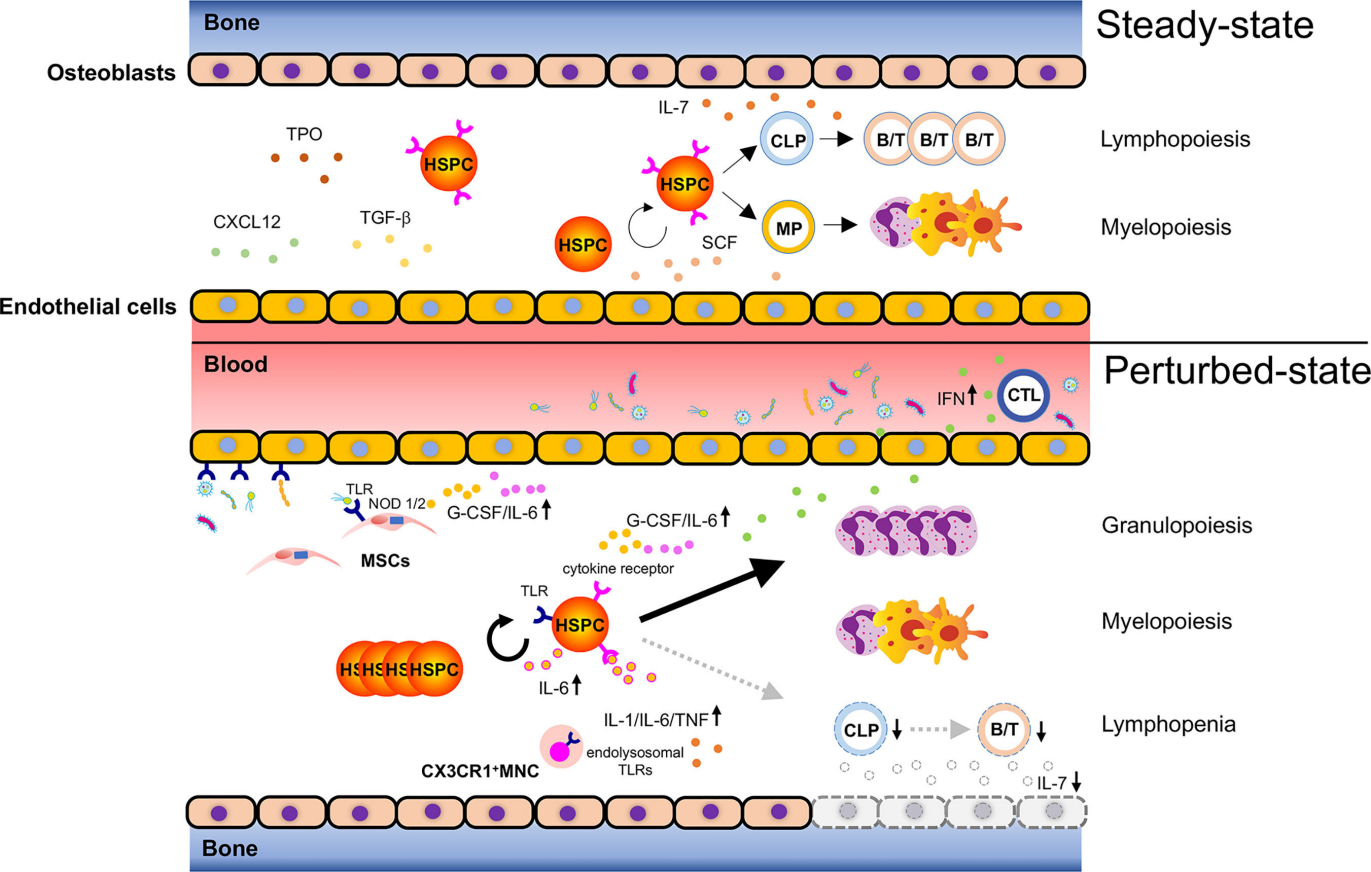
Bacteria-induced activation of HSPCs. Steady-state hematopoiesis (upper): Hematopoietic stem and progenitor cells (HSPCs) self-renew and differentiate into myeloid progenitors (MPs) and common lymphoid progenitors (CLPs) to produce mature cells. The divisional manner of HSPCs toward either self-renewal or maturation (myelopoiesis/lymphopoiesis) is tightly controlled to sustain lifelong hematopoiesis. Hematopoiesis under infection (lower): Bacterial components reach the bone marrow (BM) *via* systemic blood circulation to activate pattern recognition receptors (PRRs) such as toll-like receptors (TLRs) expressed on HSPCs and promote their proliferation. Bacteria-associated molecules reach the BM and can alternatively activate TLRs and NOD1/2 on endothelial cells or mesenchymal stromal cells (MSCs), leading to the secretion of inflammatory cytokines such as G-CSF and IL-6. These secreted cytokines promote granulopoiesis by acting on HSPCs. Cytotoxic T lymphocytes (CTLs) respond to bacterial infection and produce inflammatory cytokines such as IFNs, which migrate to the BM and activate corresponding receptors expressed on HSPCs. This results in reduced HSPC self-renewal and enhanced myelopoiesis. Severe bacterial infection such as sepsis rapidly ablates osteoblasts and induces lymphopenia due to lack of osteoblast-derived IL-7. CX3CR1^+^ mononuclear cells (MNCs) sense bacteria-derived molecules such as bacterial DNA *via* endolysosomal TLRs and secrete the inflammatory cytokines, IL-1, IL-6, and TNF, which control the expansion of hematopoietic progenitors, and shift the hematopoietic program toward myelopoiesis. Taken together, bacterial challenges induce HSPC activation and myelopoiesis directly and indirectly at the expense of lymphopoiesis.

Recent findings however highlight the dynamic response of HSCs toward inflammation. HSCs directly sense inflammation through their extracellular and intracellular receptors, rapidly lose quiescence and proliferate in response to an external milieu of inflammatory factors and infectious agents. Common inflammatory signals reported to impact primitive hematopoiesis include interferon (IFN)-α (27–29), IFN-γ (30, 31), tumor necrosis factor (TNF)-α (32), transforming growth factor (TGF)-β (33), interleukin (IL)-1 (34), IL-6 (35, 36), and macrophage colony-stimulating factor (M-CSF) (37); infectious agents include pathogen-associated molecular patterns (PAMPs) derived from microbes and danger-associated molecular patterns (DAMPs), both of which are recognized by pattern recognition receptors (PRRs). The activation of respective downstream signaling pathways in HSCs may result in their mobilization, proliferation, or differentiation to boost immune cell production (38, 39). Infection restricted to peripheral tissues/organs is primarily dealt with by immune cells at local sites that will get activated, consumed, and ultimately replenished by HSPCs (**Figure 1**). In the case of a systemic microbial spread due to severe infection or sepsis, HSPCs in the BM are activated to proliferate and drive myelopoiesis at the expense of lymphopoiesis. This is known as emergency myelopoiesis and involves the *de novo* generation and release of immature and mature neutrophils from the BM (38, 40).

Inflammatory cytokines are major regulators of stress hematopoiesis (39) (**Table 1**). Essers et al., reported that IFN-α produced by plasmacytoid dendritic cells (DCs) upon TLR9 activation (49) activated dormant HSCs and caused their entry into the cell cycle. During chemotherapy, the proliferative capacity of HSCs is likewise enhanced upon stimulation with IFN-α. The proliferative stress ensued with 5-fluorouracil (5-FU) treatment caused a profound reduction of WT HSCs, while IFNRα^−/−^ HSCs were mostly unaffected (27). IFN-γ upregulation during *Mycobacterium avium* infection similarly activated HSCs and resulted in their increased cycling and proliferation (30). However, the effect of IFNs on HSCs can be diverse, as described in the case of the tick-borne pathogen *Ehrlichia* infection, where a robust production of IFNα/β impaired hematopoiesis through HSPC depletion and enforced HSPC quiescence (50). IL-1 produced by several cell types such as macrophages, ECs, and epithelial cells (51) directly stimulated HSCs and skewed their differentiation potential toward myeloid lineages through activation of PU.1, a transcription factor regulating the myeloid differentiation program (34). Likewise, upon LPS challenge, M-CSF secreted from ECs, macrophages, or fibroblasts was found to affect PU.1, and HSCs with higher PU.1 levels were primed toward myeloid differentiation (37). TNF-α mainly secreted by macrophages, T cells, and natural killer cells (52) promotes HSC survival and simultaneously, their myeloid differentiation *via* an NF-κB-PU.1-dependent mechanism (32). An alternative pathway in response to bacterial infection, driven by intermediate lineage-committed HPCs *via* osteoblast-derived IL-7, which is a crucial cytokine for lymphopoiesis has also been reported (48).

**Table 1:** The role of inflammatory cytokines or chemokines on steady-state and stress hematopoiesis.

Cytokines	Which cells produce	Effect on HSC function	Reference
SCF	Endothelial cell, MSC	HSC maintenance	Morrison Nature 2014 (20)
CXCL12	Endothelial cell, MSC, CAR cell		
Thrombopoietin (TPO)	Hepatocyte		Decker Science 2018 (41)
Transforming growth factor β (TGF-β)	Schwann cell		Yamazaki Cell 2011 (33)
Fms-like kinase 3 (Flt-3)	Ubiquitous	Myeloid differentiation	Gabbianelli Blood 1995 (42)
Interferon (IFN)-α	Plasmacytoid dendritic cell (DC)	Impaired HSC reconstitution capacity	Esser Nature 2009 (27)
		HSC exhaustion	Sato Nat Med 2009 (28)
			Pietras J Exp Med 2014 (29)
IFN-γ	T cell	Impaired HSC reconstitution capacity	Baldridge Nature 2010 (30)
		Impaired HSC maintenance	de Bruin Blood 2013 (31)
Granulocyte colony-stimulating factor (G-CSF)	MSC, endothelial cell	Myeloid differentiation	Boettcher J Immunol 2012 (43)
			Boettcher Blood 2014 (44)
Granulocyte-macrophage colony-stimulating factor (GM-CSF)	MSC, endothelial cell, macrophage, T cell		Weisbart Nature 1985 (45)
			Shi Cell Research 2006 (46)
Macrophage colony-stimulating factor (M-CSF)	Endothelial cell, macrophage, fibroblast		Mossadegh-Keller Nature 2013 (37)
Interleukin (IL-1)	Macrophage, Endothelial cell Epithelial cell		Pietras Nat Cell Biol 2016 (34)
Interleukin (IL-3)	T cell		Suda J Cell Physiol 1985 (47)
IL-6	Ubiquitous HSPC (LSK)		Zhao Cell Stem Cell 2014 (36)
IL-7	Osteoblast	Decrease of CLPs and induction of lymphopenia	Terashima Immunity 2016 (48)
TNF-α	Macrophage, T cell, natural killer cell	Myeloid differentiation	Yamashita Cell Stem Cell 2019 (32)

The table summarizes the role of chemokines and cytokines upon steady-state or stress hematopoiesis. SCF, stem cell factor; MSC, mesenchymal stem cell; CAR cell, CXCL12-abundant reticular cell; TNF, tumor necrosis factor.

The effects of transient cytokine stimulation on HSC regulation is overall beneficial in fighting infection, but can also be detrimental when sustained by impairing HSC function as reported in the cases of chronic *Mycobacterium avium* infection (53), IFN-α (27) and LPS challenges (54), and IL-1 receptor stimulation (34). These detrimental effects may stem from the accumulation of DNA damage and double-strand breaks induced by various HSC activators (55). While the TGF-β presented by non-myelin Schwann cells is essential for steady-state HSC maintenance (33), continuous TGF-β stimulation *in vitro* appears to reduce HSC cell division and suppress their reconstitution ability (56). Interestingly, the proliferation of lymphoid-biased HSCs but not myeloid-biased HSCs, as defined by the Hoechst dye efflux efficiency or “side population” was suppressed (57). Upregulation of TGF-β was found upon *Trypanosoma cruzi* infection (58), which suggests chronic infection may also differentially impact the function of HSCs. Collectively, these findings illustrate how HSCs respond to various cytokine stimulations by adjusting their proliferative capacity as well as their differentiation program. The duration and/or magnitude of the stimulation are possible determinants for the cellular fate of HSCs (i.e., self-renewal, differentiation, or apoptosis).

Toll-like receptors (TLRs) are a family of transmembrane receptors that serve as the first-line innate immune sensor for a variety of infection-derived PAMPs and DAMPs (59). The TLRs primarily expressed on HSPCs are TLR2 and TLR4; both bind to bacterial ligands and induce their myeloid differentiation (60). Lymph-duct circulating HSPCs also express TLRs and differentiate into myeloid cells upon their ligation (61), indicating that TLR expression may serve as a means of immuno-surveillance, to sense infection at local sites and increase hematopoietic production upon need. Alternatively, the expression of bacteria-sensing receptors on HSPCs evolved to deal with life-threatening infections in the devastating case innate immune cells ever fail in combat and systemic bacterial infiltration follows. A proof-of-principle study involved an acute challenge with lipopolysaccharide (LPS), a gram-negative bacterial component recognized by TLR4, and subsequent activation of quiescent HSCs to proliferate and differentiate into myeloid cells (60–62), while upon repetitive stimulation or chronic LPS challenge, HSC numbers increase but their reconstitution capacity decrease, somewhat recapitulating HSC aging (54, 63, 64). The elicited downstream pathway appears bacterial species-dependent, as *Pseudomonas aeruginosa*, a gram-negative bacteria induced HSC expansion through TLR4 (65), whereas *Staphylococcus aureus*, a gram-positive bacteria showed similar HSC expansion but through a TLR-independent pathway (66). Live *Salmonella Typhimurium* induced proliferative stress in HSCs, albeit through TLR4-dependent and -independent mechanisms (54). Besides bacteria, the fungus *Candida albicans* also expanded HSC-containing LSK cells of the BM *via* TLR2 and prompted their differentiation into granulocytes, monocytes, macrophages and DCs (67). Of note, upon TLR2 and TLR4 activation, HSPCs were also capable of secreting IL-6, a particularly important regulator of myelopoiesis in an auto- and paracrine manner (36). Finally, sustained increase in Sca-1^+^ HSPCs is a hallmark of bacterial and viral infections as described above, but also parasitic infections as well, as demonstrated in the malaria mouse model elicited by the *Plasmodium berghei* sporozoite, *via* direct HSC and progenitor proliferation (68).

Apart from invading pathogens, commensal bacteria or the microbiota can also regulate hematopoiesis. Microbiota depletion by antibiotics pretreatment induced atrophy of the thymus and spleen, and suppressed hematopoiesis in the BM by reducing HSC, MPP, and CLP numbers in a Stat1-dependent manner, and not *via* TLR signaling (69). Similarly, germ-free (GF) mice showed lower HSC, MPP, and CLP counts, and a selective functional defect in GMP and myelopoiesis (70). This phenotype was reversible and could be rescued with administration of the nucleotide-binding oligomerization domain (NOD) 1 ligand which activated mesenchymal stem cells (MSCs) to produce the inflammatory cytokines, IL-7, IL-6, TPO, SCF, and Flt-3. These results suggest peptidoglycan (PGN), the NOD1 ligand derived from the microbiota modulates daily hematopoiesis (71). Recently, CX3CR1^+^ monocytes were found to co-localize with HSPCs near blood vessels in the steady-state BM. These monocytes sensed commensal bacteria-derived molecules *via* their endolysosomal TLRs (TLR-3, -7, and -9) to produce tonic levels of the inflammatory cytokines, IL-1β, IL-6, and TNF-α and control proliferation and myeloid differentiation of HSPCs (70). Thus, microbiota-derived molecules circulate the blood in both physiological and pathological conditions (72), reach the BM, and are captured by specific hematopoietic and non-hematopoietic cells to fine-tune hematopoiesis.

## Inflammation-Stressed BM Microenvironment

Upon tissue insult, various BM cells have been reported to influence either HSCs or hematopoiesis, including adipocytes (73), endothelial vessels (74), osteocytes (75), neurons (76), macrophages (77) and Schwann cells (33) among others. The BM microenvironment has likewise been investigated at single-cell resolution during homeostasis and under stress hematopoiesis (78). The necessity of stromal cells for efficient HSC expansion and maintenance under perturbed conditions has become apparent from studies by several groups. Co-transplantation of CD73^+^CD105^-^Sca1^+^ BM stromal cells with donor-derived HSCs after irradiation resulted in efficient repairing of the damaged niche and improved HSC expansion (79). The transplanted stromal cells were localized within clusters of HSCs, indicating the efficient expansion of HSCs following their transplantation relied on local interactions with stromal cell progenitors. Guo et al., reported the importance of Jagged-2 induction in vascular niches after myeloablation for HSPC expansion and reacquisition of HSPC quiescence (80). Recently, a subset of apelin^+^ ECs was shown to be critical not only for the maintenance of steady-state hematopoiesis but also after myeloablative injury. Apelin^+^ ECs expanded substantially and mediated the regeneration of the vascular niche and subsequent hematopoietic reconstitution after BM transplantation *via* pleiotrophin (81). Thus, reciprocal interactions between the niche and HSCs are vital in determining efficient hematopoietic reconstitution under stressed conditions.

Infection-induced HSPC activation is mediated by a combination of direct and indirect pathways involving PRRs such as TLRs and NODs expressed on hematopoietic and non-hematopoietic cells (**Tables 2** and **3**). In particular, granulocyte-colony stimulating factor (G-CSF) secreted from TLR4-expressing ECs is essential and sufficient to activate GMPs and drive emergency myelopoiesis (43). *Escherichia coli* infection rapidly mobilized HSCs to the spleen *via* two innate immune sensors, nucleotide-binding oligomerization domain (NOD)-like receptor 1/2 and TLR4, both of which are expressed on radio-resistant cells, presumably stromal cells. Their activation synergistically induced G-CSF secretion for efficient HSC mobilization and neutrophil differentiation (84). LPS challenge also drives vascular remodeling in the BM, proliferation of ECs and increase in their permeability, and accompanies HSPC proliferation and neutrophil mobilization from the BM (85). Del-1 is an extracellular matrix protein expressed by cellular components of the HSC niche, including ECs and CAR cells. Del-1 deficiency attenuated emergency myelopoiesis and HSPC expansion both in steady-state and in response to LPS and G-CSF injections (86). Intravital BM imaging revealed parasitic *Trichinella spiralis* infection dramatically increased HSC motility within the BM and their migration to other BM spaces (87). Parvovirus B19 caused transient erythroid aplasia by infecting MSCs and upregulating their expression of IL-6 and TNF-α (88). Treatment with IFN-α or pI:C, a ligand for TLR3 and a mimetic of viral infection modulates hematopoiesis *via* hematopoietic- but also niche-expressed IFN-α receptor. Both challenges increased EC proliferation in the BM partly through vascular endothelial growth factor (VEGF) (89). Taken together, these findings indicate bacterial, viral, or parasitic infections can induce HSPC activation also through niche-dependent pathways.

**Table 2:** Pattern recognition receptors expressed by hematopoietic cells that regulate steady-state and stress hematopoiesis.

Receptors	Ligands	Cell type	Species	Signaling	Function	Reference
TLR2	*Candida albicans*	LSK (Lin^−^Sca-1^+^c-kit^+^)	Mouse	TLR2-Myd88/Dectin1	Differentiation into DCs	Yanez PLoS One 2011 (67)
	Pam3CSK4	Lin^−^	Mouse	TLR2-ROS	Differentiation into macrophages with lower levels of inflammatory cytokines	Yanez Eur J Immunol 2013 (82)
		CD34^+^	Human			
TLR2/4/9	Pam3CSK4	Common dendritic cell progenitor (CDP)	Mouse	CXCR4 down-regulation and CCR7 up-regulation	DC expansion in inflamed lymph nodes and support of DC homeostasis	Schmid Blood 2011 (83)
	LPS					
	CpG					
TLR4	LPS of *Pseudomonas aeruginosa*	LSK (Lin^−^Sca-1^+^c-kit^+^)	Mouse	TLR4	Dysfunctional HSC expansion	Rodriguez Blood 2009 (65)
	LPS	HSC (CD150+CD48-LSK)	Mouse	TLR4	Increased HSC number but decreased HSC reconstitution potential	Esplin J Immunol 2011 (64)
	LPS	HSC (CD150+CD48-LSK)	Mouse	TLR4-Id1	Increased HSC number but induced HSC dysfunction	Zhao PLoS One 2013 (63)
	LPS	HSC (CD150^+^CD135^−^CD34^−^CD48^−^ LSK/CD150^+^CD34^−^CD48^−^CD41^−^ LSK)	Mouse	TLR4-TRIF- ROS-p38	Proliferative stress-induced HSC dysfunction	Takizawa Cell Stem Cell 2017 (54)
	*Salmonella Typhimurium*					
TLR3/7/9	bacterial DNA	CX3CR1^+^MNC	Mouse	TLR3/7/9	Inflammatory cytokine production by CX3CR1^+^ MNCs induced MPP expansion and steady-state myelopoiesis	Lee Blood 2019 (70)

The table summarizes the role of pattern recognition receptors expressed in hematopoietic cells upon steady-state or stress hematopoiesis. TLR, Toll-like receptor; DC, dendritic cell; Pam3CSK4, Pam3Cys-Ser-(Lys)4; ROS, reactive oxygen species; LPS, lipopolysaccharide; CCR, CC chemokine receptor; CXCR, CXC chemokine receptor; MNC, mononuclear cell; CX3CR1, CX3C chemokine receptor 1; HSC, hematopoietic stem cell; MPP, multipotent progenitor.

**Table 3:** Pattern recognition receptors expressed by non-hematopoietic cells that regulate steady-state and stress hematopoiesis.

Receptors	Ligands	Cell type	Signaling	Species	Function	Reference
TLR4	LPS	Non-hematopoietic cell	TLR4 not IL-1R	Mouse	G-CSF-mediated emergency myelopoiesis	Boettcher J Immunol 2012 (43)
		Endothelial cell	TLR4-Myd88			Boettcher Blood 2014 (44)
TLR4	LPS	Non-hematopoietic cell	G-CSF up-regulation and CXCL12 down-regulation	Mouse	G-CSF-induced HSC mobilization to spleen	Burberry Cell Host Microbe 2014 (84)
NOD1/2	PGN					
NOD1	PGN	MSC	NOD1	Mouse	Regulation of steady-state hematopoiesis *via* cytokine production by MSCs	Iwamura Blood 2017 (71)

The table summarizes the role of pattern recognition receptors expressed in non-hematopoietic cells upon steady-state or stress hematopoiesis. TLR, Tol- like receptor; LPS, lipopolysaccharide; G-CSF, granulocyte-colony stimulating factor; NOD, Nucleotide-binding oligomerization domain-containing protein; PGN, peptidoglycan; MSC, mesenchymal stem cell.

## Chemotherapy- and Irradiation-Induced Inflammation

Similar to naturally occurring infections, quiescent HSCs are recruited to actively divide and regenerate the hematopoietic system in response to artificial BM ablating agents, such as irradiation or chemotherapy. Here, HSC activation is likely caused by a transient surplus of systemic cytokines that occur after irradiation- or chemotherapy-induced BM suppression. Cytokine levels in the serum or BM, including SCF and TPO were elevated due to their reduced consumption by surrounding hematopoietic cells (90, 91). Specifically, the elevation of TPO, SCF, IL-3, FLT3, and CXCL12 after lethal irradiation protected HSPCs from apoptosis and improved the survival of irradiated mice (92). Other cytokines of note include TNF-α, IL-1β, and IL-6 detected in the serum (93) and additionally IL-1α, IFN- α/β, and GM-CSF in several cell types (94). Thus, not only local but also systemic cytokine levels determine the fate of HSCs post BM suppression.

Low mitochondrial membrane potential in steady-state HSCs is maintained by extracellular adenosine supplied by surrounding myeloid progenitors, known to possess an anti-inflammatory effect (95). After 5-FU administration, the ablation of surrounding myeloid progenitors will result in low adenosine and consequently high mitochondrial activity and reactive oxygen species (ROS) production in HSCs. This is essential for initiating HSC cellular division and hematopoietic repopulation (95), but contradicts with a previous study claiming the negative regulation of ROS on HSC maintenance (96), and high mitochondrial membrane potential with reduced hematopoietic repopulating ability in steady-state and IFN-α-stimulated HSCs (97). Thus, the response of activated HSCs toward ROS is context-dependent and markedly differs from quiescent HSCs in steady-state. This is further evidenced in HSCs with enhanced mitochondrial activity and ROS levels possessing more potential for rapid regeneration of Mks and platelets after 5-FU administration (98). Here, TPO injection enhanced mitochondrial activity in HSCs and primed their differentiation toward the Mk-lineage. Not only are Mks a rich source of inflammatory cytokines and chemokines released upon acute injury and inflammation, but they can also cooperate with neutrophils to trap invading pathogens (99, 100). Among the cytokines, C-X-C motif ligand 4 (CXCL4) (22) has been reported to increase hematopoietic recovery of 5-FU-induced BM suppression (101). Fibroblast growth factor 1 (FGF1) supplied by Mks also contributed to the expansion of HSCs after chemotherapeutic stress by counteracting TGF-β inhibitory signaling (23). Furthermore, Mks help expand endosteal niche-residing osteoblasts after irradiation through the secretion of platelet-derived growth factor (PDGF)-BB, and thereby support hematopoietic recovery (102). Taken together, HSC-generated Mks and platelets serve as an essential source of hematopoietic recovery factors that regulate HSCs directly and indirectly through the BM niche.

Since BM injury induced by irradiation or chemotherapy affects not only hematopoietic cells but also non-hematopoietic cells within the BM, the reconstruction of the niche may well be key for a successful hematopoietic recovery. Angiopoietin-1 supplied from osteoblasts protects HSCs from BM suppression (103). Of note, angiopoietin-1 secreted by LepR^+^ stromal cells can also negatively influence hematopoietic regeneration after irradiation (104). Adipocytes are an additional BM niche component found to proliferate extensively after irradiation or chemotherapy to promote hematopoietic regeneration by supplying SCF to HSCs, which under normal circumstances is provided by LepR^+^ cells and ECs (73). Other factors besides SCF, including adiponectin (105) and leptin (indirectly *via* adipogenesis) can support HSC proliferation after irradiation (106). Moreover, a radio-resistant CD105^-^CD73^+^NGFR^hi^ stromal subset expressing high levels of hematopoietic cytokines was found to support hematopoietic regeneration after irradiation (107). Co-transplantation of MSCs overexpressing PDGF-β improved the engraftment of transplanted HSCs *via* enhanced HSC self-renewal and expansion (108). Niche regeneration precedes HSC regeneration after irradiation, and by enlarging the niche pool, a better environment to facilitate HSC engraftment can be achieved. Thus, BM niches are also affected by BM injury, and these alterations contribute to the regeneration of the hematopoietic system.

Since irradiation or chemotherapy damages the DNA of hematopoietic cells, molecules relevant for the DNA damage and repair machinery play a key role in hematopoietic recovery. For instance, histone deacetylase 8 (HDAC8), which modulates p53 activation contributes to HSC survival by blocking apoptosis. HDAC8-deficiency showed hematopoietic failure and increased lethality after the administration of 5-FU (109). Similarly, deficiency of the growth arrest and DNA-damage-inducible protein (Gadd45a), a key tumor suppressor showed efficient recovery of the hematopoietic system through enhanced proliferation of HSPCs, although at the expense of their genomic integrity (110). These phenotypes are attributed to a decrease in HSPC apoptosis due to a greater resistance to DNA damage. Additionally, deficiency of Rap1, a member of the shelterin proteins decreased double-strand DNA break repair through the non-homologous end-joining pathway, and consequently HSC survival after irradiation or chemotherapy (111). Thus, the ability of HSCs to respond efficiently to DNA damage is one of several factors that determines HSC survival under stress conditions. However, since excess resistance to DNA damage will increase the risk for pathogenesis, particularly in the case of Fanconi anemia (55) and leukemogenesis (110), an appropriate balance is required for a healthy hematopoietic recovery.

After BM injury, HSCs and their niches respond to a damaged BM environment by calling for an alternate response compared to steady-state conditions, which may continuously adjust until a return to quiescence. These serial changes may well dictate the appropriate and balanced supply of stem cells and differentiated cells, and the efficient regeneration of the hemato-immune system and BM niche. Further studies are needed to clarify such possibilities.

## Immune-Memory in HSPCs

Host immune responses can be divided into a rapidly reacting innate response that is relatively non-specific, and a slowly developing adaptive response that is highly specific to the antigens of invading pathogens. After clearance of an infection, the latter can form a type of immunological memory, ensuring a swift and robust response against future infections and lifelong immune protection. The concept of immunological memory was restricted to adaptive immunity but has since been extended to include innate immunity in the last decade. Indeed, various innate immune cells (i.e., monocytes, macrophages, natural killer (NK) cells) show a long-term adaptation of increased reactivity upon secondary stimulation, a state termed trained immunity (112). Epigenetic reprogramming such as histone modifications and chromatin reconfigurations established during a previous challenge is the basis for trained immunity. Upon stimulation with the TLR2 ligand β-glucan, epigenetic profiling of monocyte to macrophage differentiation has been especially revealing in terms of trained immunity signatures (113). β-glucan pre-exposed macrophages produced more inflammatory cytokines such as TNF-α and IL-6 after a secondary challenge with tripalmitoyl glyceryl cysteine, a TLR2 ligand compared to naive macrophages. In contrast, LPS pre-exposure induced immune-tolerance in macrophages. Epigenetic marks in the promoter (ACp1) and distal elements (Ace1) of H3K27ac were altered by β-glucan, whereas LPS exposure induced changes in a small subset of the dynamic distal regulatory elements (Ace5) of H3K27ac. A shift in cellular metabolism is also a key driver of trained immunity, as is the case for β-glucan trained monocytes, from oxidative phosphorylation to aerobic glycolysis *via* the mTOR-HIF1α pathway (114). This metabolic switch may enable rapid cytokine and metabolite production to combat intruding pathogens and is not restricted to glycolysis but also glutaminolysis, accumulation of fumarate (115), the mevalonate pathway and cholesterol synthesis (116).

**Figure 2:**
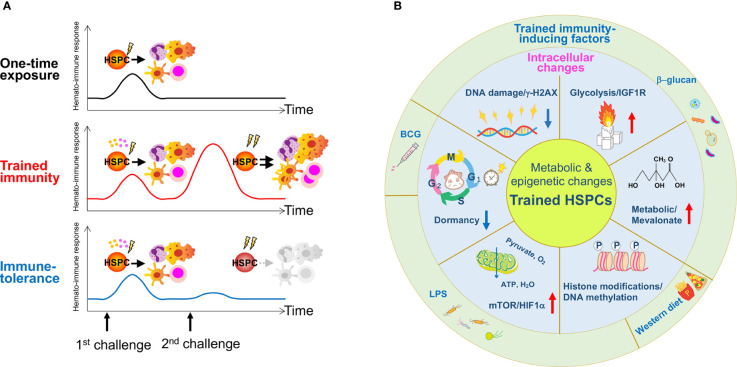
The concept of trained immunity in HSPCs and reported stressors for its induction. **(A)** A one-time exposure to an immunological challenge drives proliferation and differentiation of HSPCs to enhance host immunity. A primary challenge by innate immune insults such as BCG, β-glucan or a Western-type diet induces epigenetic or metabolic changes at the cellular level in HSPCs, and activates them directly *via* cell intrinsic changes or indirectly *via* cytokine production such as IL-1β and GM-CSF. A secondary challenge such as LPS re-stimulation enhances overall immune response, cytokine production and myelopoiesis (trained immunity). Due to memory formation, HSPCs respond better to a secondary challenge and produce more reactive immune cells that can exert robust immune responses against the infection. A hypothetical scheme of immune-tolerance is shown. Immune-tolerance induces immune suppression upon a secondary challenge, which impairs HSPC function and their potency to differentiate into myeloid cells. As a result, immune responses decline and renders the host more susceptible to infection. **(B)** The schematic figure summarizes findings published in previous reports and highlights the role of inflammation on trained HSPCs. Several types of inflammation-causing components including β-glucan, a Western-type diet and BCG affect HSPCs at the intracellular level. These factors induce metabolic and epigenetic changes such as enhanced glycolysis and cholesterol biosynthesis, histone modifications, changes in cell cycle state and an increase in DNA damage.

The paradox that short-lived myeloid lineages (monocytes and DCs) with a turnover of every 5 to 7 days retain trained immunity features lasting several months to years served as a motive for investigating long-lived HSPCs and their potential to be trained (117). Recent studies report trained immunity in HSPCs after acute/chronic stimulation by inflammatory cytokines and pathogen-derived agents, such as LPS, β-glucan, or BCG (**Figure 2A**). Relevant epigenetic, metabolic and key signaling pathways that activate or exhaust stem cell activity will be described here (**Figure 2B**).

β-glucan stimulation induced expansion of the myeloid-biased CD41^+^ HSC and MPP subset of the BM that persisted well after transplantation (117). A metabolic shift toward enhanced glycolysis, the mevalonate pathway and cholesterol synthesis in HSPCs was observed. Initial exposure to β-glucan enhanced the response of HSPCs to a secondary systemic LPS challenge by expansion of the LSK and MPP population and an elevated DNA damage response (lower γ-H2AX levels) (117). Kaufmann et al., similarly reported cell expansion and enhanced myelopoiesis of BCG-educated HSCs and MPPs in the BM *via* an IFN-γ-mediated pathway. These trained HSCs generated epigenetically-modified macrophages with better protection against *Mycobacterium tuberculosis* infection (118). Primary LPS stimulation likewise elicited trained immunity in HSCs, enabling a faster and more robust response against a secondary *Pseudomonas aeruginosa* infection (119). Here, a single LPS challenge induced HSC expansion 1 day post injection, which returned to homeostatic levels within 5 days. Transcriptome analysis revealed significant gene expression changes 1 day post injection but a return to normal expression patterns after 4 weeks. Analysis of chromatin opening/closing sites showed newly created open chromatin regions in HSPCs (CD135^-^LSKs) relevant for the immune response and myeloid differentiation, which were retained 4 weeks post injection. These open chromatin regions were significantly lost in HSCs deficient in TLR4 and C/EBPβ, a mediator of TLR signaling, and demonstrated LPS-TLR4-C/EBPβ-mediated epigenetic memory formation in HSCs (119). Thus, trained immunity confers a protective outcome on HSPCs and ultimately host defense by enhancing the reactivity of mature cells derived from “trained HSPCs” toward a secondary infectious challenge.

Constitutive stimulation by inflammation or infection can also result in immune-tolerance, a state of reduced reactivity (**Figure 2A**). LPS pre-exposure induced immune-tolerance in monocytes by inactivating epigenetic marks in lipid metabolism and phagocytosis-related genes; these were partially reverted upon β-glucan stimulation (120). Similarly, BCG vaccination induced H3K4me3 activation in human monocytes *via* the upregulation of IL-1β, a key mediator of trained immunity (121). Given that HSPCs chronically stimulated by IL-1β prior to transplantation showed compromised hematopoietic regeneration possibly due to immune-tolerance (34), the induction of trained immunity or immune-tolerance may depend on cell type and/or the duration of the signal, and possibly metabolic adaptability. Recently, a “sterile” Western-type diet was reported to trigger trained immunity in the *Ldlr* ^−/−^ atherosclerosis mouse model through proliferation and functional reprogramming of GMPs into activated and potentially harmful monocytes (122). Hypercholesterolemia reprogramming of HSCs has also been described (123, 124). Thus, the relevance of immune-tolerance is readily implicated in chronic inflammatory diseases and possibly for the treatment of certain autoimmune diseases such as type 2 diabetes.

## Effect of Immune Insults on Hematologic Malignancies

Hematopoietic homeostasis is perturbed when the immune system is challenged, such as in cases of infection or inflammation. A systemic increase of inflammatory cytokines and chemokines will stimulate effector immune cells, stromal cells and HSPCs to rapidly replenish consumed innate immune cells at peripheral sites. Stress-induced cell cycle activation of quiescent HSCs will boost hematopoiesis to restore tissue homeostasis, but may come with dire consequences if chronically sustained (i.e., chronic inflammation and autoimmune diseases). Chronic immune stimulation induces cell stress, DNA damage and various hematopoietic dysfunctions, as observed in patients with sickle cell disease often developing myeloid malignancies, possibly from the associated cytokine storm that can cause somatic gene mutations and myeloid neoplasms (125). Persistent cytokine or PAMP stimulation *via* TNF, IFN, and IL-6 signaling and HSC dysfunction have been well-documented and may readily impact the initiation and progression of hematologic malignancies and BM failure (126).

The risk for developing hematologic malignancies increases exponentially with age (127–129). Although not a hematopoietic challenge per se, aging displays a chronic inflammatory phenotype often associated with expansion of the HSC compartment and lineage-bias toward myeloid and megakaryocytic cells (130). The increase in inflammatory factors, IL-6, IL-1, and C-reactive protein accompanies aging (131) and is basis for the emergence of inflammaging (132). As reported by Mann et al., aged and young HSCs display contrasting responses toward inflammatory stress (133). A myeloid-biased subset, which expands with age and are further marked by CD61 expression shows a poor response to prolonged infectious challenges, and possibly are prone to myeloid leukemia development.

Closely associated with aging is clonal hematopoiesis of indeterminate potential (CHIP), a precursor state where mutations in leukemia-associated driver genes are acquired in individuals with no prior history of hematologic diseases, and thus posing a neoplastic progression risk (134). Somatic genes with high potential to develop into hematopoietic malignancies upon mutation include epigenetic modifiers, splicing factors, proliferation signaling molecules and DNA-damage regulators such as DNMT3A, TET2, ASXL1, JAK2, SF3B1, PPM1D and TP53, all known to be mutated in prominent hematologic malignancies (135, 136). Clonal hematopoiesis is a predictor state with adequate potential toward the development of hematologic malignancies (137), while the indeterminate potential aspect of the name reflects the uncertainty behind why only a small population of individuals displaying clonal hematopoiesis develop into full-blown leukemia. Specific mutations may augment inflammation and drive HSC proliferation, while the inflamed environment may further foster genetic ablations in some HSCs and result in their selective expansion. Indeed, several patient studies report of cases where inflammatory conditions promote clonal hematopoiesis (138, 139), and illustrate how HSC impairment upon inflammatory stress may provoke their malignant transformation. This possibility is supported by epidemiological evidence where a history of infection/autoimmunity strongly correlates with hematologic malignancies (140, 141).

A prominent driver mutation in hematological neoplasms is TET2. A recent study reported the abnormal expansion of myeloid cells in *Tet2*-deficient mice (142). Cull et al., found LPS induced *Tet2* transcription in macrophages, while *Tet2* loss enhanced the secretion of the pro-inflammatory cytokines, IL-6, IL-1β, and TNF and the expression of LPS-induced genes associated with an inflammatory state (143, 144). This alteration toward an inflammatory environment may favor *Tet2*-mutant HSPC expansion (145). Interestingly, Meisel et al., reported a breach in the intestinal barrier and subsequent translocation of bacteria result in increased IL-6 production. The risk for development into a pre-leukemic myeloproliferation state was heightened in mice with *Tet2*-deficinent expression in hematopoietic cells, which was similarly recapitulated in *Tet2*-deficient germ-free mice upon colitis induction or in response to systemic bacterial stimuli such as treatment with a TLR2-agonist (146). This study highlights the requirement for microbial-dependent inflammation in the development of pre-leukemic myeloproliferation.

TLR signaling is also essential for the inflammatory response by shaping HSC fate and blood cell output and if dysregulated, contributes to the loss of HSC potential and/or their malignant transformation (147). Recently, aberrant TLR signaling and its downstream effector molecule, Myd88 has been linked to myelodysplastic syndrome (MDS) and acute myeloid leukemia (148–150). As HSCs express several TLRs enabling their direct stimulation, a causal link between innate immune signaling, HSC dysfunction and hematologic malignancies can be readily imagined, as supported by the following studies. The expression of TLR2 and TLR4 genes was found higher in patients with myelomonocytic and monoblastic acute leukemia (151). Huang et al., reported enhanced innate immune response pathways in chronic myeloid leukemia mouse models (152). MDS patients were found to overexpress TLR1, TLR2, TLR4 and TLR6 in human CD34^+^ cells (153, 154). Activation of the NF-κB pathway contributed to HSPC apoptosis in MDS, possibly *via* a family of Nod-like receptors (NLRs) and inflammatory-mediated cell death, or pyroptosis. Particularly, the NLR protein 3 (NLRP3) inflammasome overexpressed in MDS HSPCs increased secretion of IL-1β and IL-18, and caused pyroptotic cell death and eventual cytological dysplasia (147, 155, 156). An inflamed environment (i.e., chronic inflammation) was found to promote MDS progression by providing MDS HSPCs with a competitive advantage over normal HSPCs. The mechanistic basis for their clonal dominance occurred *via* a switch from canonical to noncanonical NF-κB signaling in TLR-TRAF6 primed HSPCs that ultimately sustained myeloid expansion (157). A novel perspective designating the BM niche as the driving force for the initiation and evolution of MDS pathogenesis has been elaborated upon in another review (158) Thus, inflammation is a key determinant for the competitive advantage of MDS HSPCs over normal HSPCs.

Abnormal activation of autoreactive T cells and a shortage in stem cells have been reported in both aplastic anemia (AA) patients and mouse models as the ruling cause of BM failure and appears central to the pathophysiology of acquired AA (159). Increased CD4^+^ helper T cells and activated CD8^+^ cytotoxic T cells can be found in the patient’s BM and are suspected as culprits in HSPC and BM destruction (160). The CD4^+^ T cells dominant in acquired AA secrete IFN-γ and TNF-α, and have been reported to inhibit CD34^+^ colony formation. The adenylate-uridylate–rich element (ARE)-deleted mouse model that constitutively expresses IFN-γ, revealed IFN-γ alone could disrupt CMP generation, prevent hematopoietic differentiation and recapitulate AA pathology (161). Apart from AA, the most often inherited bone marrow failure syndrome, Fanconi anemia is associated with defective DNA repair and genomic instability, which are also primary hallmarks of aging. In addition to pI:C injections, Walter et al., demonstrated the ability of other physiological stimuli (i.e., IFN, G-CSF, TPO or serial bleeding) to cause DNA damage in LT-HSCs *in vivo* within similar ranges of pI:C, enforce HSC exit out of quiescence, and accelerate failure of the hematopoietic system as observed in Fanconi anemia patients (55). These findings collectively illustrate a causal link between HSC dysfunction induced by chronic immune stimulation and progression toward hematopoietic failure and hematologic malignancies.

## Discussion

Since the initial establishment of the hematopoietic differentiation tree, our understanding of the hematopoietic system, and of the HSC population situated at its apex undergoes continuous refinement. Formerly presumed unresponsive to tissue insult, HSCs in fact show high adaptability under various scenarios and actively cooperate with downstream hematopoietic progenitors, mature cells, and environmental stromal cells as frontline responders to preserve blood homeostasis. However, their ability to respond deftly through self-renewal and differentiation at times brings about detrimental consequences. In this review, we sought to address the latest understanding of HSC biology, in terms of heterogeneity, functionality, and adaptability in steady-state versus perturbed conditions with a particular emphasis on infectious and inflammatory challenges.

From ontogeny to aging, the functional readout of a single HSC in terms of repopulation ability and lineage output differs immensely, leading to the concept of clonality and heterogeneity. Only recently through the development of single-cell approaches can we now address the most basic questions: How many HSCs are born during definitive hematopoiesis formation (162)? Are all HSCs identical in terms of lineage fate (7, 15)? Do all HSCs equally contribute to daily hematopoiesis? Are HSC responses equal under perturbed conditions (133)? The list of questions is ever-expanding. However, transplantation-based studies to test for HSC functionality, where the recipient is subjected to irradiation is a non-physiological setting and should be interpreted with caution as the readout reflects lineage potential enforced on a single HSC rather than its native fate. For example, expanding HSCs after 5-FU treatment contain elevated ROS levels due to high mitochondrial activity but also high repopulating ability (95) and contrasts with steady-state HSCs, where high mitochondrial activity normally implies reduced repopulating ability (96). HSCs can perhaps change their cell fate, depending on the surrounding environment. However, whether this reflects genuine HSC heterogeneity or simply activation of an emergency program remains unresolved. The implementation of new technology to assess lineage output in unperturbed states, such as the inducible sleeping beauty transposon system enabling barcoding of single cells and lineage reconstitution by sequencing (4, 163) or the HUE mouse system (164) is beneficial here. Of note, the definition of HSCs (i.e., long-term, short-term and differences amongst the MPP subset) is still ambiguous and their exact contribution to steady-state hematopoiesis remains controversial. Different conclusions may thus be drawn depending on the experimental system at hand and must be examined carefully.

It is now clear that the BM is not immune-ignorant but a prominent lymphoid organ that receives a large spectrum of hemato-immunological insults. Likewise, BM-residing HSCs are not just quiescent sleeping cells but directly respond to insults not limited to infection, inflammation but also the regeneration of the BM after toxic agents or irradiation. Depending on the type of DAMPs, PAMPs, cytokines and growth factors involved and the strength/duration of the stimulation, HSCs will alter their fate toward myelopoiesis, granulopoiesis or even bypass progenitors altogether to directly orchestrate on-demand hematopoiesis. HSCs positioned at the interface of perturbed hematopoiesis will execute distinct emergency programs to integrate and fine-tune responses to maintain hematopoietic integrity. However, such beneficial effects of HSC activation can be counteracted by chronic inflammatory conditions. HSC dysfunction upon chronic inflammation or aging as the cause of clonal hematopoiesis and in certain cases leukemic transformation are all readily imaginable scenarios, although direct causality remains to be demonstrated. Emerging reports of trained immunity in HSPCs and mature cells derived from “trained” progenitors with an enhanced protective function provide a novel opportunity for interpretation. The well-established immune response against inflammatory or infectious stimuli may have well been under the influence of HSC trained memory and should be revisited. How HSC memory is formed and maintained, and to what degree trained immunity in HSCs dictate host immune defense are areas yet to be explored. Whether the metabolic shift in HSCs induced by memory formation alters the depth of HSC quiescence, population hierarchy and functional heterogeneity, and ultimately clonal hematopoiesis are primary but still unresolved questions. Despite the risk for potential collapse of the hematopoietic system, HSCs nonetheless persist at the frontline not only as an integrative hub for incoming inflammatory signals, but also execute tissue repair in organs beyond the blood system. Trained immune memory in HSCs offers one more additional perspective in elucidating the true nature of HSCs.

In line with this, counterpart immune/stromal niche components also regulate steady-state and stress hematopoiesis. The importance of stromal cells as a major source of HSC maintenance and activation factors for HSC homeostasis is clear. As observed by the apelin^+^ EC subset tasked to regenerate the BM after irradiation (81), ECs are highly heterogeneous in terms of their identity and function. Other BM constituents, including adipocytes that proliferate and secrete SCF post irradiation to promote BM regeneration (73), certain MSC progenitors that maintain both lymphoid progenitors and HSCs *via* CXCL12 (165), as well as a subset of regulatory T cells with high CD150 expression that localize in HSC niches and maintain HSC quiescence are additional examples of HSC interaction with immune/stromal heterogeneity (166). Thus, the heterogeneity of HSCs and their counterpart niche cells become vital when interpreting stressed conditions such as inflammation, infection and the onset of hematologic malignancies. Nonetheless, the bigger question would be, whether this so-called heterogeneity of HSCs (among others) is a distinct population or a continuum where cells retain the ability to transform back and forth. Single-cell RNA-sequencing, despite its immense power offers only a snapshot analysis and may not reflect the true nature of these cells.

Finally, the recently proposed concept of immune memory in HSPCs is a prime topic with clinical relevance. Only several studies till now have demonstrated trained immunity at the level of hematopoietic progenitors *via* β-glucan, LPS and BCG, and more can be expected. Besides transcriptional and epigenetic reprogramming and a metabolic shift as key characteristics of trained immunity, much remains to be revealed. For example, the similarities and differences in trained immunity between different stimuli, or whether the formation of a trained memory is mutually exclusive or synergistic. Regarding the duration of trained immunity as well as the cellular/molecular mechanisms associated with it, whether the stability of different signatures, e.g., chromatin modification, histone/DNA methylation, RNA splicing impact the half-life of the memory formed is a primary question among others. Furthermore, what determines whether a cell is able to form a memory? Is memory a privilege granted only to the hematopoietic compartment or do niche/stromal cells possess this ability as well and does this affect their interaction with HSCs? Although yet to be demonstrated, this possibility can readily be imagined as certain stromal cells also take part in the immune response by secreting inflammatory cytokines and chemokines and express PRRs. BCG vaccination induced trained immunity in human monocytes *via* IL-1β (121), whereas IL-1β re-stimulation damaged the repopulation ability of HSCs post transplantation (34). The latter situation may imply the induction of immune-tolerance, and a possible relevance with aging-associated HSC dysfunction due to IL-1 upregulation observed in the elderly (131). It is important to understand the determinants for dictating trained immunity versus immune-tolerance, and whether the type of stimuli or threshold of signal strength or duration determines the choice for one over the other. Lastly, trained immunity may possibly have detrimental outcomes, as in instances of autoimmune diseases (167), so can HSC memories be a predisposition for future hematopoietic malignancies, say in terms of CHIP progression to MDS? More studies are expected in the near future.

## Author Contributions

All authors studied the literature and wrote the manuscript. All authors contributed to the article and approved the submitted version.

## Funding

This work was supported in part by KAKENHI from Japan Society for the Promotion of Science (JSPS) (20K17381 to YH, 19H03688 to TU), KAKETSUKEN (to YH and TU), JSPS fellowship (201820690) and a grant for Excellent Graduate Student at Kumamoto University to MS, and JSPS (18H02843 and 18K19520), The NOVARTIS Foundation, Yasuda Memorial Foundation, and Center for Metabolic Regulation of Healthy Aging at Kumamoto University to HT.

## Conflict of Interest

The authors declare that the research was conducted in the absence of any commercial or financial relationships that could be construed as a potential conflict of interest.
